# Influence of a circular obstacle on the dynamics of stable spiral waves with straining

**DOI:** 10.1038/s41598-022-18602-0

**Published:** 2022-08-25

**Authors:** Devanand Jaiswal, Jiten C Kalita

**Affiliations:** 1 Complex Systems and Dynamics Group, Indian Institute of Technology Madras, Chennai, 600036 India; 2 Department of Mathematics, Indian Institute of Technology Guwahati, Guwahati, 781039 India

**Keywords:** Applied mathematics, Computational science, Biological physics

## Abstract

The current study envisages to investigate numerically, probably for the first time, the combined effect of a circular obstacle and medium motion on the dynamics of a stable rotating spiral wave. A recently reconstructed spatially fourth and temporally second order accurate, implicit, unconditionally stable high order compact scheme has been employed to carry out simulations of the Oregonator model of excitable media. Apart from studying the effect of the stoichiometric parameter, we provide detailed comparison between the dynamics of spiral waves with and without the circular obstacles in the presence of straining effect. In the process, we also inspect the dynamics of rigidly rotating spiral waves without straining effect in presence of the circular obstacle. The presence of the obstacle was seen to trigger transition to non-periodic motion for a much lower strain rate.

## Introduction

Numerical and experimental studies of pattern formation are of great interest in a wide variety of diverse mathematical, biological, chemical, and physical systems. However, in the last few decades, significant amount of progress has been made in understanding the distinguishing features of patterns found in its sub-domain, spatially distributed excitable media^1–12^. One of the most captivating patterns found in the two-dimensional excitable media is that of rotating spiral waves. Such spiral waves have been ascertained in the notable oscillatory Belousov–Zhabotinsky (B–Z) chemical reaction, named after two Russian scientists Boris Belousov and Anatol Zhabotinsky^7,8,13–15^. The B–Z reaction is described as the autocatalytic oxidation of an organic acid (originally malonic acid) by potassium bromate. It is quite intriguing that rotating spiral waves also exist in the heart muscle (cardiac tissue) in an abnormal cardiac rhythm, and this phenomenon is commonly known as cardiac arrhythmia^1,16–19^. These spiral waves are thought to be the result of re-entrant excitation of myocardial cells. Moreover, spiral wave patterns have also been encountered in other biological systems such as slime mold Dictyostelium discoideum^20^, plankton population dynamics^21^, etc.

Initially, it was believed that spiral waves present in the B–Z reaction rotate periodically only, until Winfree^7,8^ through his rigorous inspection, found out that spiral waves present in the B–Z reaction could also exhibit non-periodic rotations. For such non-periodic rotating spiral waves, he coined the term “meandering”. The periodic and non-periodic rotations which are typical of the spiral waves, have found their application in the field of medical research as well. They have been profoundly used in the demonstration of the ECG (electrocardiographic) patterns obtained during both monomorphic and polymorphic cardiac arrhythmias, as well as in ventricular fibrillation^1,9,22,23^. In contrast, it has been concluded through many clinical studies^1,17,18,24–27^ that the monomorphic ventricular tachycardia (VT) and polymorphic ventricular tachycardia (VT) may correspond to stable and meandering (or drifting) spiral waves, respectively. Further, they also observed that ventricular fibrillation (VF), which is often preceded by ventricular tachycardia, may correspond to spiral wave break-up leading to the formation of multiple spiral waves that are continuously eliminated and recreated. It is noteworthy that ventricular fibrillation, which is believed to be the most common cause of sudden cardiac death, appears to be the most complex representation of spiral waves^17^.

Owing the above reasons, numerous numerical and experimental investigations have been carried out in order to probe the complex features of spiral waves dynamics^1,8,10,11,28–33^. Most of the earlier theoretical results are obtained by solving the coupled nonlinear reaction-diffusion models such as Barkley^11,30^, FitzHugh-Nagumo^34^, Oregonator^10,35^, etc. The general form of these models can be written as

1 $$\begin{aligned} \frac{\partial U(x,y,t)}{\partial t}&=\frac{1}{\varepsilon }F(U(x,y,t),V(x,y,t))+D_U\nabla ^2U(x,y,t), \end{aligned}$$

2 $$\begin{aligned} \frac{\partial V(x,y,t)}{\partial t}&=G(U(x,y,t),V(x,y,t))+D_V\nabla ^2V(x,y,t). \end{aligned}$$

Here, *U*(*x*, *y*, *t*) and *V*(*x*, *y*, *t*) are the excitable (fast) and recovery (slow) variables respectively with corresponding diffusion coefficients $D_U$ and $D_V$. *F* and *G* are the nonlinear reaction terms associated with the variables *U* and *V* respectively. The parameter $\varepsilon$ is a constant such that $0<\varepsilon <1$.

The spiral tip plays a crucial role in understanding the dynamics of a spiral wave as it evolves, which can be explored by tracing the movements of the spiral tip. Interestingly, despite the crucial role played by the spiral tip, almost all previous numerical studies employed the state variables (e.g.^31,34,36^) to examine the transition of spiral waves from periodic to nonperiodic motions, particularly the phase-portrait analysis. The authors^28,29^, in their recent publications have explored the dynamics of the spiral wave by the sole use of tip paths exploiting the power spectra analysis through Fast Fourier Transformation. We had investigated the transition of a spiral wave from periodic to nonperiodic rotation in an excitable media and their dependence on the reaction parameters by using the Barkley^28^ and Oregonator^29^ models.

It is worth noting that the anatomical obstacles such as blood vessels, scars tissues are inherently present in the myocardial tissues (an excitable medium), which can greatly alter the dynamics of spiral waves^1,27,37–39^. For example, in the study of Kim *et al.*^38^, a meandering or drifting spiral wave when pinned to an obstacle, was seen to attain stability and persistence in the long run. This phenomenon is very similar to activities like ventricular fibrillation switching to ventricular tachycardia regime^1,38^. The recent experimental, theoretical and numerical studies in^1,40–42^ reported that pinning of spiral waves effectively depends on the size of obstacles. Moreover, the interaction between a meandering spiral wave and an obstacle may also result in spiral wave break-up^39^, which can be mapped into the occurrence of ventricular fibrillation. However, almost all the earlier numerical studies on spiral wave dynamics in presence of obstacle are concerned only about the pinning or unpinning of the spiral waves^27,40,41,43–46^. Further, in their recent article, Jaiswal and Kalita^28^ have also demonstrated the effect of a square obstacle on a rigidly rotating spiral wave by placing it outside the core region. They performed numerical simulations through the Barkley model and concluded that the core region gets displaced from its original position which results in a change of the trajectory of the spiral tip that eventually settles into a periodic motion again.

The medium motion plays a significant role in the dynamics of spiral waves^2,4,29,47,48^. For example, experiments with the B–Z reaction in laboratory have revealed that the introduction of strong convective motion of chemically reacting medium not only triggers chaos but also results in the break-up of the spiral wave^10^. Interestingly, a similar chaotic phenomenon leading to spiral break-up is also considered to be the most likely mechanism underlying ventricular fibrillation^1,26^. Note that the cardiac muscle itself is another relevant example of a moving excitable medium as the propagation of excitation waves may depend on the muscle’s motion^2,4,49^. Despite the crucial role of medium motion in spiral dynamics, the lion’s share of all earlier numerical studies had been performed assuming the excitable medium to be at rest. One of the ways of handling the effect of medium motion mathematically is to introduce convective terms in system (1)–(2) for both variables *U* and *V*. In their investigation of the dynamics under the effect of one-dimensional shear flow, Biktashev and his group^2,4,49^ have concluded that it can result in distortion and break-up of spiral waves. Ramos^47,48^ and most recently, Jaiswal and Kalita^29^ have studied the effect of medium motion by incorporating the two-dimensional shear flow in the Oregonator model of excitable media and concluded that spiral wave deforms and stretches under the influence of straining. This stretching and deforming was seen to generate a host of complex patterns in the spiral waves.

The current study endeavours to investigate some hitherto unexplored regime of rotating spiral wave dynamics by probing the effect of a circular object inside the core region of a stable spiral wave in an exciting medium with motion. This is opposed to the earlier studies where the obstacle placed in the excitable media was outside the core region^28^. To the best of our knowledge, for the first time, the influence of a circular obstacle on the dynamics of the stable rotating spiral wave is being investigated by considering the effect of straining (medium motion) at the same time. Once again tracking the spiral tip over an extremely large time range occupies the core of our analysis and was seen to unfold several new features of the physiological phenomena. All numerical simulations reported in the study are performed through the Oregonator model^29^ of the reaction-diffusion system, by exploiting a recently developed implicit, unconditionally stable, temporally second and spatially fourth order accurate high order compact (HOC) finite difference scheme^28^.

This article has been organized in the following way. In the next “Model and method” section, we provide a brief description of the Oregonator model and the numerical method in use. Then in subsequent “Effect of a circular obstacle on the dynamics of spiral waves in the absence straining” section, explore the effect of the circular shaped obstacle in the absence of straining, while “Effect of obstacle in the presence of straining” section deals with the impact of the obstacle in the presence of straining. Finally, in “Conclusions” section, we conclude our achievements.

## Model and method

In order to describe the temporally and spatially oscillatory complex structures arising out of the B–Z chemical reaction, Field *et. al.*^50^ derived the mathematical model involving the concentrations of three intermediates, which is widely known as the Oregonator model. Following their works, Tyson *et. al.*^51^, have further modified their model, which only has two concentrations *u* and *v* of the intermediates now. The spatially extended form of the Oregonator model^35^, can be written as:

3 $$\begin{aligned} \frac{\partial u}{\partial t}&=\frac{1}{\varepsilon }\left( u-u^2-av\frac{u-b}{u+b}\right) +D_u\left( \frac{\partial ^2 u}{\partial x^2}+\frac{\partial ^2 u}{\partial y^2}\right) , \end{aligned}$$

4 $$\begin{aligned} \frac{\partial v}{\partial t}&=u-v+D_v \left( \frac{\partial ^2 v}{\partial x^2}+\frac{\partial ^2 v}{\partial y^2}\right) . \end{aligned}$$

which can be obtained from the general form (1)–(2) by substituting for the functions *F* and *G*. Here, reaction parameters *a*, *b*, and $\varepsilon$ are positive constants, and their values depend on the reaction kinematics. Notably, *a* is a stoichiometric factor that can be adjusted and alter the dynamics of the excitation waves by changing its values^35^. The parameter *b* has an extremely small magnitude, $0< b<< 1$ and is not affected by the chemical concentrations. $D_u$ and $D_v$ are the diffusion coefficients corresponding to *u* and *v* respectively.

Due to the presence of the highly nonlinear first term on the right hand side of (3), analytical solution of the coupled R–D system (3)–(4) is extremely difficult to find. As such, one must resort to numerical computation of its solutions. A thorough inspection of the earlier numerical studies^1,6,9,10,24,30,31,39,47,48^, reveals that most of them have been accomplished by exploiting explicit, lower-order accurate, and conditionally stable finite difference methods such as the forward time and centered space (FTCS). Therefore, in order to discretize the system of equations (3)–(4), we employ the recently devised implicit, unconditionally stable, temporally second and spatially fourth-order accurate high order compact (HOC) finite difference scheme in^28^. In the following, we briefly describe the numerical scheme.

The two-dimensional unsteady R–D equation for the transport variable $\phi (x,y,t)$ may be formulated as:

5 $$\begin{aligned} \frac{\partial \phi }{\partial t}-D\nabla ^2 \phi +d(x,y,t) \phi =f(x,y,t). \end{aligned}$$

Assuming the spatial domain $\Omega$ to be of rectangular shape given by $\Omega = [x_{\mathrm{min}},x_{\mathrm{max}}] \times [y_{\mathrm{min}},y_{\mathrm{max}}]$ and generating the discrete points $(x_i,y_j)$ by the intersection of vertical and horizontal lines given by

$$x_i=x_{\mathrm{min}} +ih_x, \;\;\;\ y_j=y_{\mathrm{min}}+jh_y, \;\;\;\; i=0,1,2,...,I_{\mathrm{max}}-1 \;\;\;, \text {and} \quad j=0,1,2,...,J_{\mathrm{max}}-1.$$

**Figure 1:**
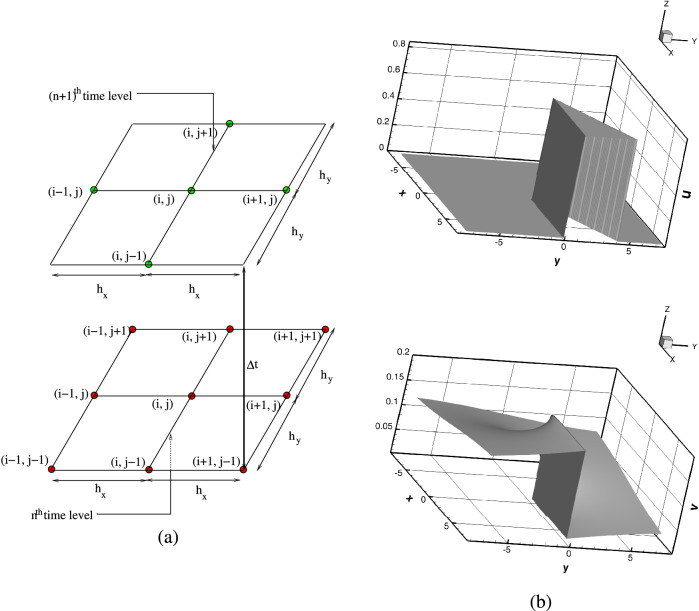
(**a**) Unsteady High Order Compact stencil for the R–D equation and (**b**) surface plots of the initial profiles of *u* and *v*.

The uniform step lengths along *x*- and *y*-directions are defined as $h_x = {\frac{x_{\mathrm{max}}-x_{\mathrm{min}}}{(I_{\mathrm{max}}-1)}}$ and $h_y ={\frac{y_{\mathrm{max}}-y_{\mathrm{min}}}{(J_{\mathrm{max}}-1)}}$ respectively. Using a uniform time-step $\Delta t$, the $O((\Delta t)^2, h_x^4,h_y^4)$ HOC scheme for (5) is given by

6 $$\begin{aligned}&\left[ 1-\frac{D \Delta t}{2}\left( \delta _x^2+\delta _y^2\right) +\left( \frac{h_x^2}{12}\delta _x^2+\frac{h_y^2}{12}\delta _y^2 \right) +\frac{\Delta t}{2}d_{ij}^n\right] \delta _t^+\phi _{ij}^n -\frac{D\left( h_x^2+h_y^2\right) }{12}\delta _x^2\delta _y^2\phi _{ij}^n \nonumber \\&\quad +\left[ \left( \frac{h_x^2d_{ij}^n}{12}-D\right) \delta _x^2+\left( \frac{h_y^2d_{ij}^n}{12}-D\right) \delta _y^2+E_{ij}^n\right] \phi _{ij}^n +\left[ \frac{h_x^2}{6}\delta _x d_{ij}^n\delta _x+\frac{h_y^2}{6}\delta _y d_{ij}^n\delta _y\right] \phi _{ij}^n\nonumber \\&=G_{ij}^n, \end{aligned}$$

where $E_{ij}^n=\left( 1+\frac{h^2}{12}\delta _x^2+\frac{k^2}{12}\delta _y^2+\frac{\Delta t}{2} \delta _t^-\right) d_{ij}^n, \; G_{ij}^n=\left( 1+\frac{h^2}{12}\delta _x^2+\frac{k^2}{12}\delta _y^2+\frac{\Delta t}{2} \delta _t^-\right) f_{ij}^n$. Here $\delta _x$, $\delta _x^2$, $\delta _y$, $\delta _y^2$ are the first and second order central difference operators in the spatial directions *x*- and *y*- respectively, and $\delta _t^+$ and $\delta _t^-$ are the forward and backward difference temporal operators. The stencil for (6) requires nine points at the $n\mathrm{th}$ and five points at the $(n+1){th}$ time level as shown in Fig. 1a.

Numerical simulations are carried out by discretizing the system of equations (3)–(4) by (6) as (3)–(4) can easily be put in the form (5) by slight adjustment of the terms. For example, by setting $u=\phi$, $d=-1/\varepsilon$, $D_u=D$ and $f=-\frac{1}{\varepsilon }\left( u^2+av\frac{u-b}{u+b}\right)$, one can obtain (6) from (3). Likewise for (4). The spatial domain has been chosen as the square $[-7.5,7.5]\times [-7.5, 7.5]$, which has further been discretized into $241 \times 241$ points so that $h_x=h_y=\frac{1}{240}$ and a uniform temporal step length $\Delta t=10^{-4}$ throughout all the computations. At the boundaries, zero flux conditions are imposed. The initial conditions are set as:

$$\begin{aligned}&u= {\left\{ \begin{array}{ll} 0.8,&{} \text {if } 0< \theta <0.5\\ b\frac{a+1}{a-1}, &{} \text {otherwise} \end{array}\right. }\\&v= {\left\{ \begin{array}{ll} b\frac{a+1}{a-1}+\frac{\theta }{8\pi a}, \end{array}\right. } \end{aligned}$$

where $\theta$ is the angle (radians), measured counter-clockwise from the positive *x*-axis with respect to origin. The surface plots of the initial profiles for *u* and *v* are shown in Fig. 1b.

Throughout all the computations, the value of parameters are set as: $b=0.002$, $\varepsilon =0.01$, $D_u=1.0$ and $D_v=0.6$, while *a* was considered in the range $1.4 \le a \le 1.72$. The time step for all numerical simulations is set as $10^{-4}$, and the initial conditions for *u* and *v* are the same as depicted in Fig. 1b^29,47^. Furthermore, the location of the spiral tip (denoted by $(x^*,y^*)$), at a particular instant of time has been obtained by finding the intersection of isocontours $u=u_{tip}$ and $v=v_{tip}$. While value of $u_{tip}=0.15$ is fixed, the value of $v_{tip}$ is obtained by solving $f(u_{tip},v_{tip})=0$, where $f(u,v)=(1/\varepsilon ) \left( u-u^2- av(u-b)/(u+b) \right)$. For more details about how the tip position $(x^*,y^*)$ has been computed numerically, one can refer to the section 3.1.3 of the authors’ work in^28^.

## Results and discussion

### Effect of a circular obstacle on the dynamics of spiral waves in the absence straining

**Figure 2:**
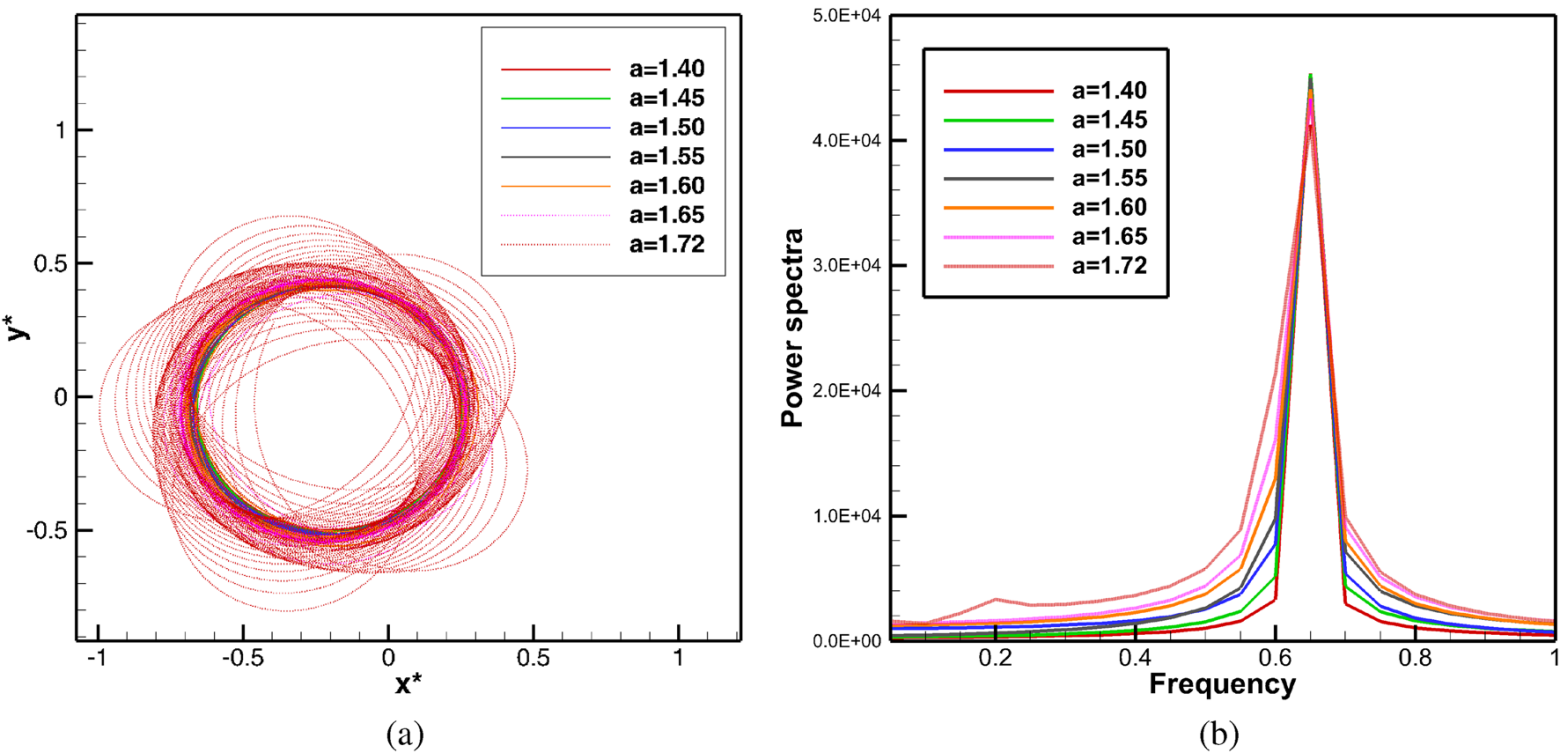
(**a**) Tip trajectories and (**b**) power spectra of the *x*-coordinate of the tip trajectories of the spiral waves for $1.40\le a \le 1.72$ in the time range $10\le t\le 100$.

**Figure 3:**
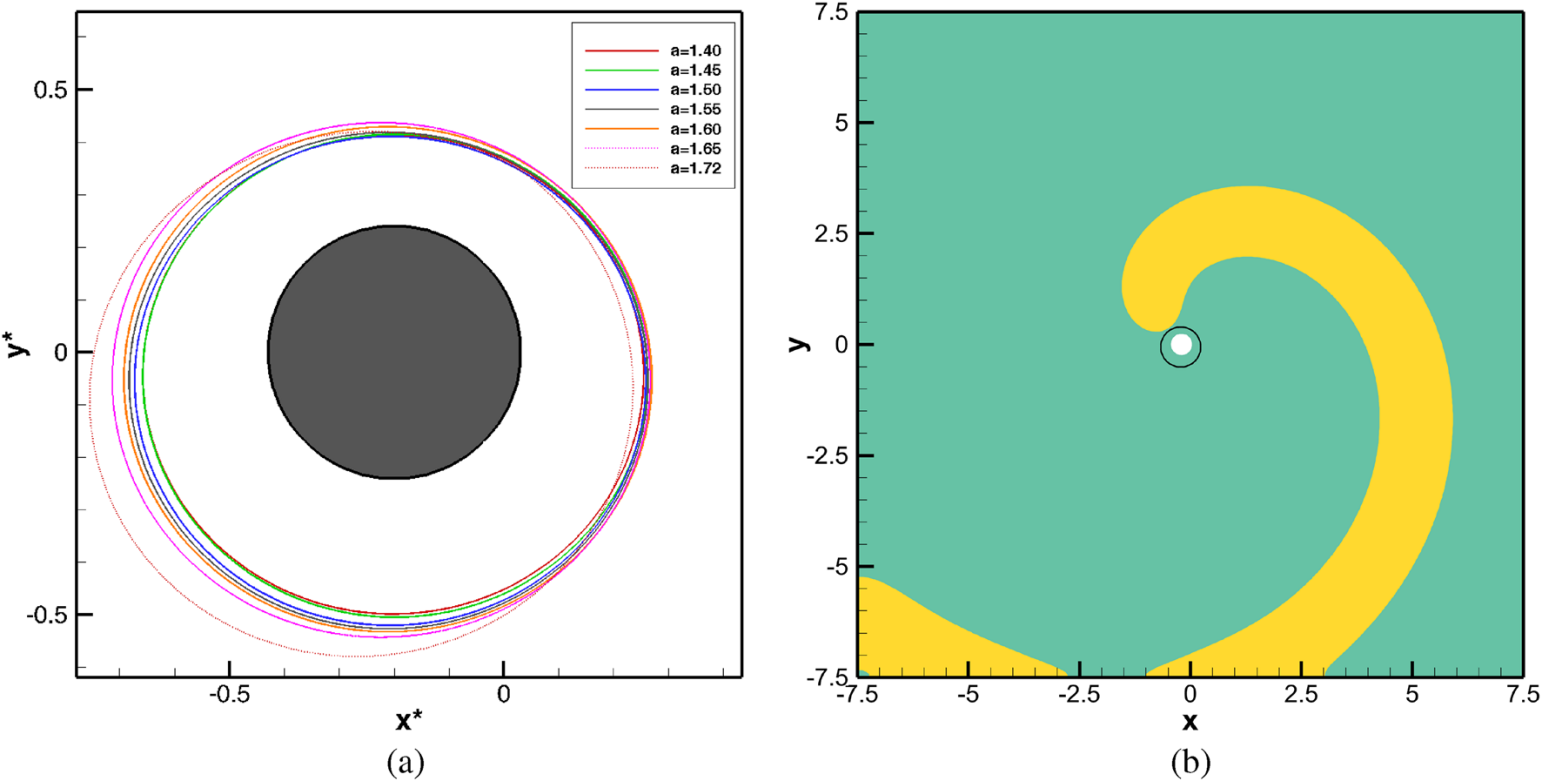
(**a**) The spiral cores for $1.40 \le a \le 1.72$ captured in the time range $90 \le t \le 100$ and the circular obstacle, and (**b**) a sample spiral wave pattern at $t=100$ just at the onset of the obstacle being placed when $a=1.72$.

Firstly, simulations were performed when neither any obstacle nor medium motion was present in the domain under consideration by solving equations (3)–(4) in its original form. For the whole range $1.4 \le a \le 1.72$, the spiral waves were seen to settle into a periodic motion within a very short span of time $t \le 100$. The spiral tip paths eventually curve out circles of varied diameters as can be seen from Fig. 2a corresponding to the time range $10 \le t \le 100$ (see the enlarged tip paths in Fig. 3a for a more precise view in the time range $90\le t\le 100$). These periodically settled circular orbits define the core region. The associated power spectra analysis of the time history of *x*-coordinate of the tip paths in Fig. 2b reconfirms the periodicity of the spiral wave motion as only one dominant mode of frequency can be observed here. Note that the power spectra of the *x*- and *y*-coordinates are identical for all the cases. Interestingly, the diameters of these circles (shown in Fig. 3a) increase with increase in the values of the parameter *a*. Since all values of *a* correspond to the same dominant frequency value (Fig. 2b) 0.65001, one can conclude that the spiral waves move faster as *a* increases.

Next, we examine the effect of a circular obstacle on the periodic rotating spiral waves in the absence of straining. It may be mentioned that in our earlier study^28^, we had investigated the effect of square obstacles on stable rotating spiral waves by placing it outside of the core region. On the other hand, the current study deals with the impact of a circular obstacle when it is inside the core region.

Having established the periodicity of the spiral waves at $t \le 100$, and also using the fact that the location of the spiral cores have been confirmed for all $1.4 \le a \le 1.72$, we now place a circular obstacle in this core region as in ^41^. In actual computation, it is a group of discrete points lying inside the small disk defined by $D_{\delta }(x_0,y_0)=\{ (x,y) \in R \;|\;\sqrt{(x-x_0)^2+(y-y_0)^2}\le \delta \}$, where *R* is the square domain $[-7.5,7.5] \times [-7.5,7.5]$. For those interested in the effect of the size of the obstacle, may refer to the recent theoretical analysis proposed by Gao et al.^42^ for the existence of a minimal obstacle. The point $(x_0,y_0)$ and $\delta$ are chosen in such a way that the obstacle always lies inside the circles (core regions) traced by the tip trajectories for all values of $1.4\le a \le 1.72$ (see Fig. 3a). We choose $(x_0,y_0)=(-0.2,0.0)$ and $\delta =0.23$ in our computations. Thenceforth, we investigate the temporal evolution of spiral waves by tracing the tip movement, and for this, simulations are performed till $t=1500$. For the benefit of the readers, we plot a sample spiral wave pattern corresponding to $a=1.72$ along with the circular obstacle (the white disc) is in Fig. 3b at the instant $t=100$ when it is placed inside the core (the circle with black boundary). This circular obstacle is mathematically modelled by assuming the values of variables $u=0$ and $v=0$, on and inside the circle.

**Figure 4:**
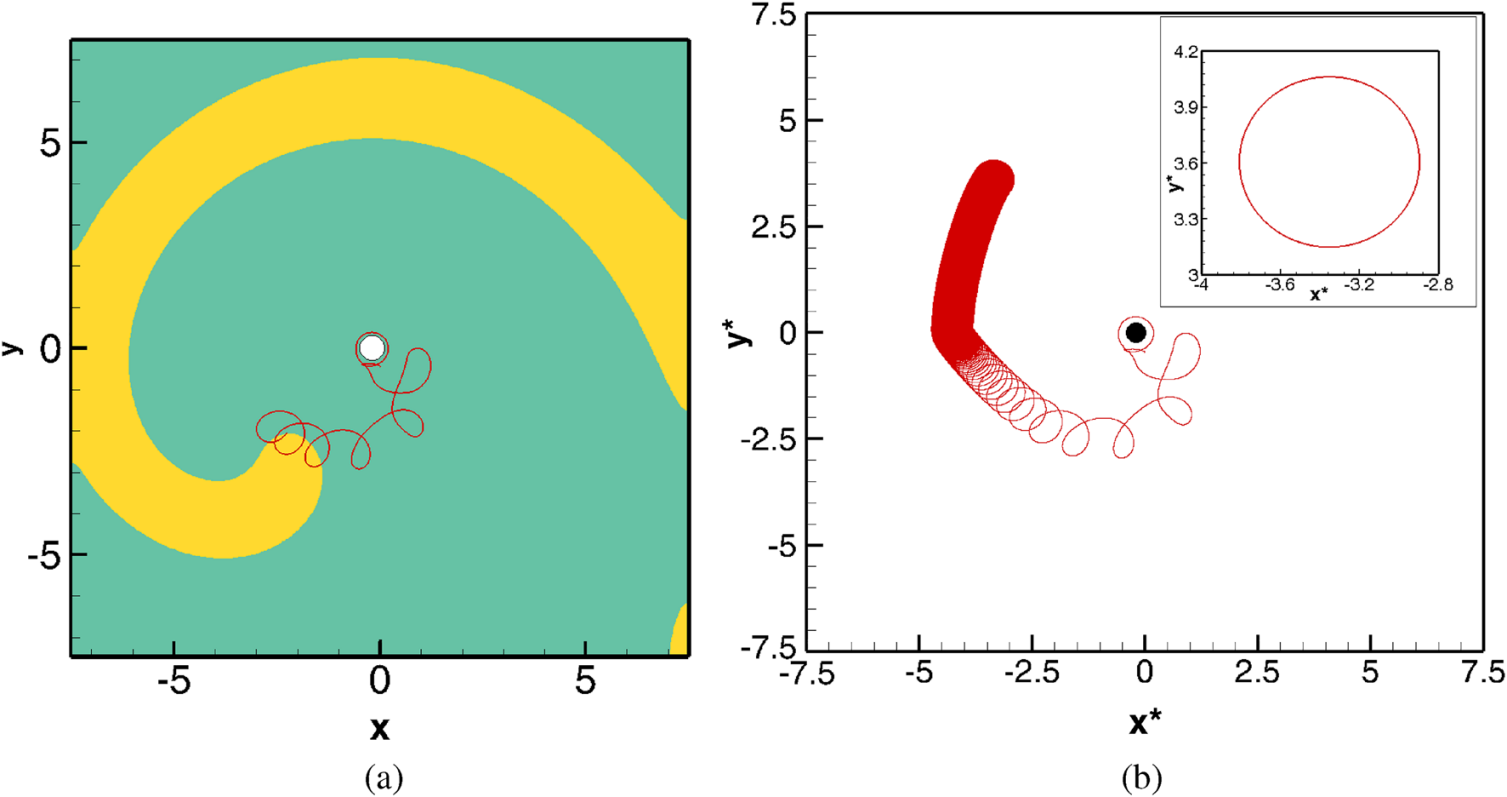
Dynamics of spiral wave in the presence of a circular obstacle for $a=1.40$: (**a**) the tip trajectory during $100\le t\le 110.00$, along with spiral wave pattern at $t=110$, (**b**) tip trajectory for $100.00\le t\le 1500.00$ along with the one during the final time range $1450\le t\le 1500$ at the inset.

The effect of placing the obstacle inside the spiral core for $a=1.40$ can be seen from Fig. 4a, b. In Fig. 4a, we show the spiral wave at $t=110$ along with the tip path in time range $100\le t \le 110$. Though initially the spiral wave was seen to revolve around the circular obstacle, after completing one revolution, which was achieved at $t=101.40$, in sharp contrast to what had happened in absence of the obstacle (Fig. 2a), the spiral tip starts drifting gradually away from the circular obstacle. It follows the trajectory as illustrated in Fig. 4b and eventually settles into a periodic motion at a later time. This can be clearly seen in its enlarged version presented at the inset of the same figure, where the trajectory of the tip in the $x^*y^*$- phase plane is shown corresponding to $1450\le t \le 1500$. The trajectory being a closed curve confirms the periodicity of the spiral wave.

**Figure 5:**
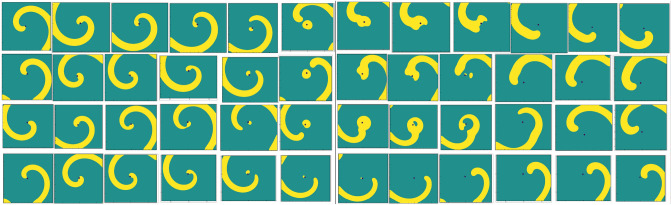
Earliest evolution of the spiral waves for in the presence of the circular obstacle (blue spot) for $101.1 \le t \le 103.0$ for $a=1.45$ (top row), $a=1.50$ (second row), $a=1.60$ (third row) and $a=1.72$ (bottom row) showing the spiral wave front hitting the obstacle, breaking up and then merging once again.

**Figure 6:**
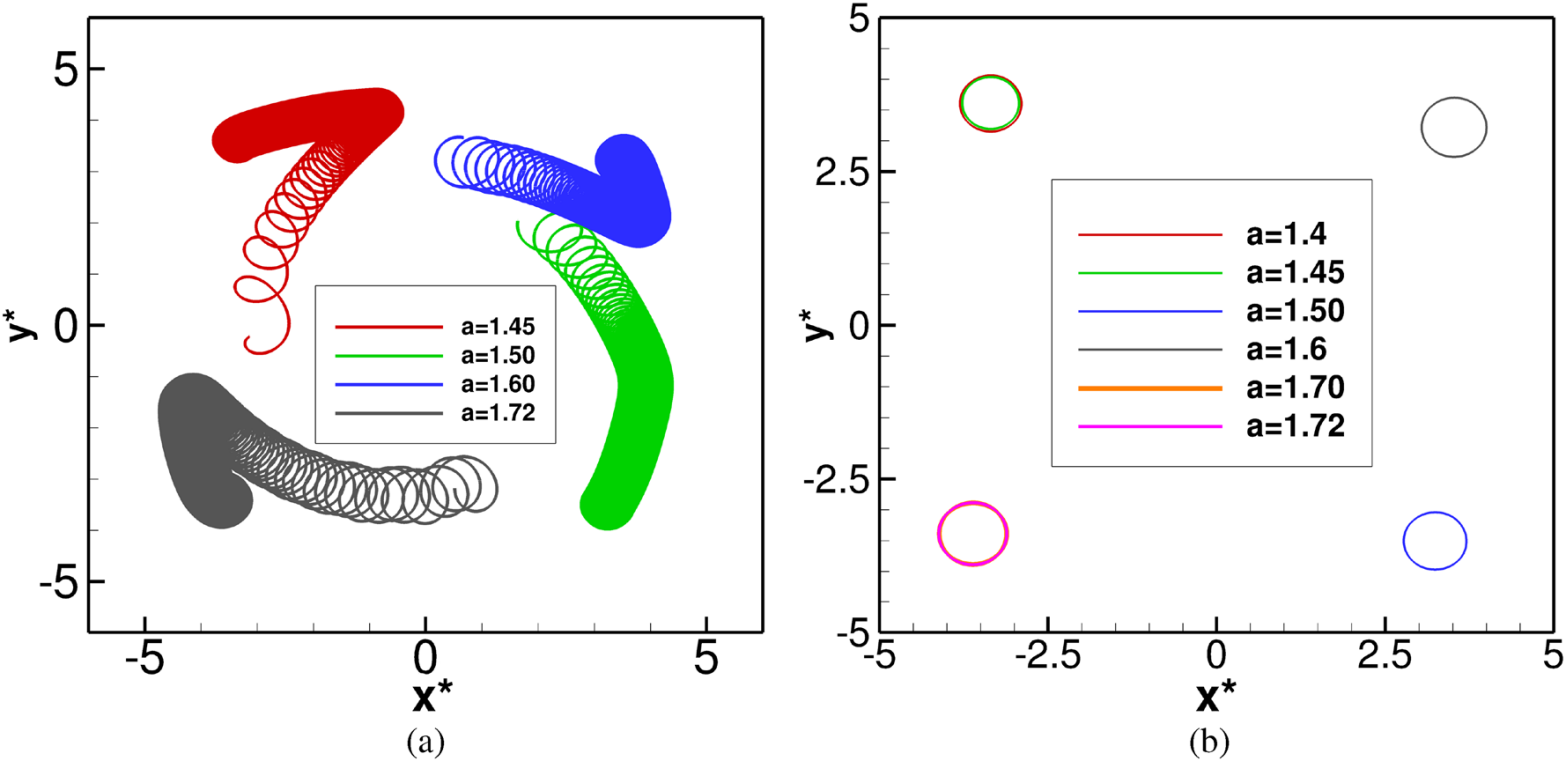
Dynamics of spiral wave in the presence of a circular obstacle: (**a**) tip trajectories for $a=1.45,\; 1.50,\; 1.60\; \mathrm{and}\; 1.72$ in the time range $110.000\le t\le 1500.00$, and (**b**) displaced core regions of the spiral waves for different values of parameter *a*.

Next, we increase the value of the parameter *a* and carry out simulations till $a=1.72$ by following the same procedure as in $a=1.4$ mentioned above. Contrary to the movements of the spiral waves for $a=1.4$, a striking phenomenon is observed within a very short span of time just after placing the obstacle for all the values of $a>1.4$ considered in this study. Instead of revolving around it at the beginning, the wave fronts hit the obstacle, break up and then merge again and continue to drift along. We illustrate this phenomenon in Fig. 5 where the evolution of the spiral waves during $100.1 \le t \le 103$ are depicted in twelve successive snapshots for $a=1.45,\; 1.5,\; 1.6 \; \mathrm{and} \; 1.72$ in each rows. After that, the spiral tips were seen to drift from the core region once again. Note that it is not possible to track the wave tips corresponding to the time span of Fig. 5 as the tip tracking algorithm fails to do so. As such, we have shown the tip trajectories from $t=110$ till $t=1500$ in Fig. 6a where one can see them finally settling into periodic motions once more. However, the trajectories followed completely different directions depending upon the value of the parameter *a*. For example, for $a=1.4, 1.45$ they settle into circular paths in the north-west, for $a=1.5, 1.55$ south-east, $a=1.6, 1.65$ north-east and for $a=1.70, 1.72$, the south-west directions away from the obstacle.

**Figure 7:**
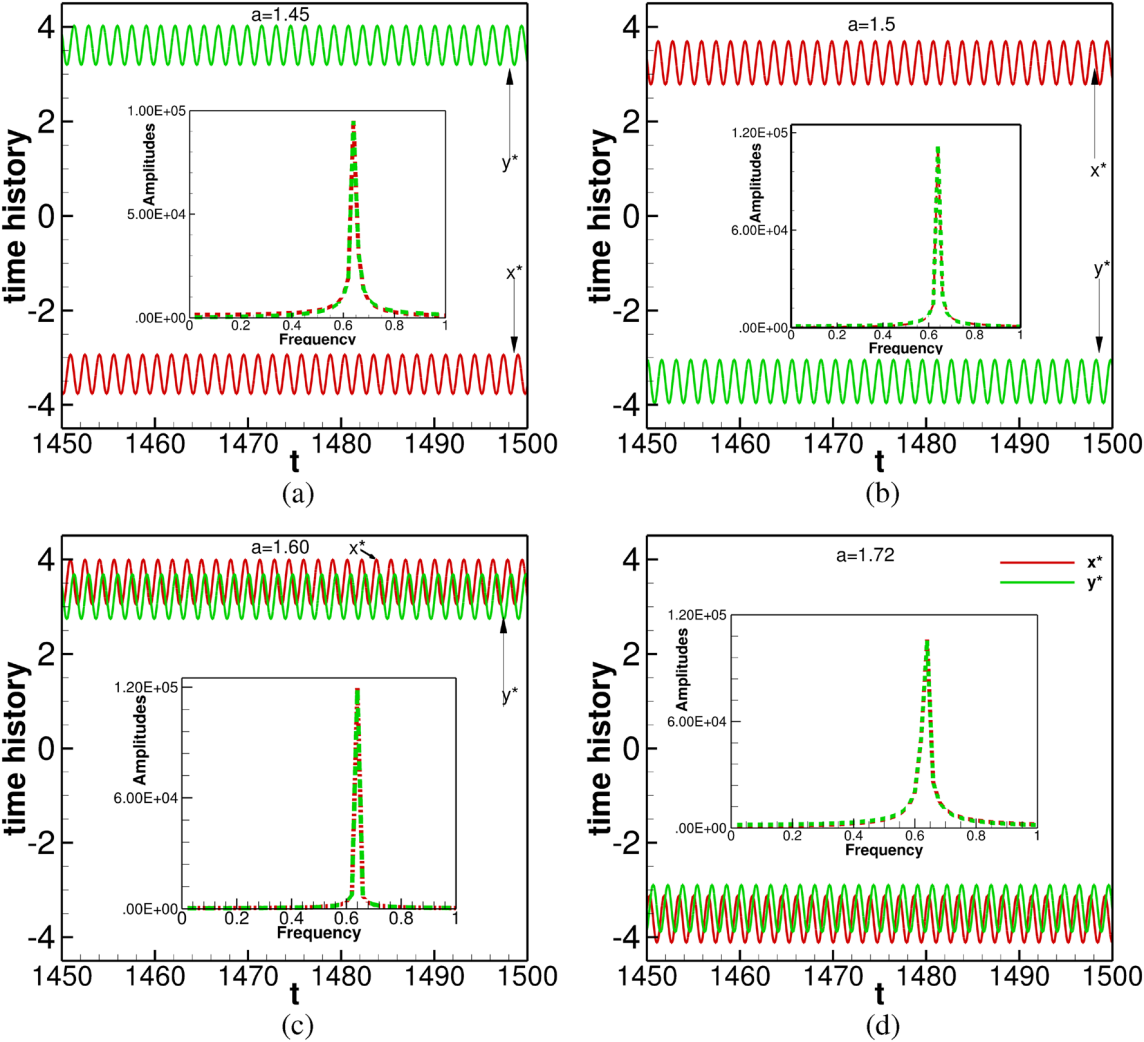
Time history plots of tip coordinates $(x^*,y^*)$, and their corresponding power spectra in small frames (inset), with obstacle for *a*: (**a**) 1.45, (**b**) 1.50, (**c**) 1.60 and (**d**) 1.72.

The trajectories finally settling into circular orbits at different corners of the computational domain for $1450\le t \le 1500$ are shown in Fig. 6b. In order to get more insights into the periodic behaviour of the of spiral waves as $t \rightarrow \infty$, we have plotted time histories of tip paths $(x^*,y^*)$, in Fig. 7 for different values of $1.4\le a \le 1.72$ in the time range $1450\le t\le 1500$ along with their respective power spectra at the inset. The direction of the tips can also be gauged from the signs of the *x* and *y* coordinates of the tips. Expectedly, the spectra for all the values of *a* yield a single dominant mode. Interestingly, this mode occurs at the frequency value 0.65001 for all $1.4\le a \le 1.72$ designating a time period of rotation $=1.538$ for each of them. More significantly, this is exactly similar to the frequency and time periods for the spiral waves without the presence of an obstacle documented in the previous section. Thus, for the range of the parameters considered here, the role of the obstacle was to change the trajectory paths and create new core regions for the spiral waves.

### Effect of obstacle in the presence of straining

Now, we embark on investigating the effect of a circular obstacle on the dynamics of periodically rotating spiral waves in exciting media when straining (medium motion) is present. Mathematically, this is accomplished by adding some advection terms into the system (3)–(4) for the variables *u* and *v*. Consequently, the system (3)–(4) takes the form:

7 $$\begin{aligned} \frac{\partial u}{\partial t}&=\frac{1}{\varepsilon }\left( u-u^2-av\frac{u-b}{u+b}\right) +D_u\left( \frac{\partial ^2 u}{\partial x^2}+\frac{\partial ^2 u}{\partial y^2}\right) -\mathbf{V}_\mathbf{e}.\nabla u, \end{aligned}$$

8 $$\begin{aligned} \frac{\partial v}{\partial t}&=u-v+D_v \left( \frac{\partial ^2 v}{\partial x^2}+\frac{\partial ^2 v}{\partial y^2}\right) -\mathbf{V}_\mathbf{e}.\nabla v. \end{aligned}$$

Here, $\mathbf{V}_\mathbf{e}=(V_e^x, V_e^y)$ is the velocity vector whose components corresponding to the coordinates directions *x* and *y* are $V_e^x$ and $V_e^y$ respectively, with $\nabla$ being the two dimensional gradient operator. The velocity components are given by $V_e^x=-\gamma y$ and $V_e^y=-\gamma x$, where the constant $\gamma$ is the strain rate^29^. This irrotational $(\nabla \times \mathbf{V}_\mathbf{e}=0)$ and solenoidal $(\nabla . \mathbf{V}_\mathbf{e}=0)$ velocity field has been chosen in such way that the compressible effect is not instigated by the convection terms, and as such the reaction rate remains unaffected^29,47,48^. In the actual B–Z reactions, introduction of the advection terms can also be considered as equivalent to the application of an external electric field, which can directly affect the dynamics of spiral waves by causing convective motion of ionic species in reaction^40,52^.

While (3)–(4) represented a system of R–D, with the introduction of $\gamma \ne 0$, (7)–(8) is now a system of unsteady convection-reaction-diffusion equations. By appropriately adjusting the terms present in this system, one can easily apply the HOC scheme (6) developed for R–D equations to discretize (7)–(8). The authors’ work^29^ is a testimony to that. Interested readers may refer to^29,53^ for more details on how one can solve (7)–(8) by converting it into a system of purely R–D Equations. Once again the simulations have been performed on a grid of size $241\times 241$, with zero flux boundary conditions at all the boundaries of the square domain $[-7.5, 7.5]\times [-7.5, 7.5]$. Further, the value of parameters $a=1.4$, $b=002$, $\varepsilon =0.01$, $D_u=1.0$ and $D_v=0.6$ have been kept fixed throughout this section.

In the recent work^29^, authors investigated the dynamics of a rigidly rotating spiral wave under the effect of straining with no obstacle present in the domain of the study. Since our current work is concerned about the dynamics of stable rotating spiral waves under the effect of a circular obstacle in the presence of medium motions of the excitable media, it will be worthwhile to mention briefly the observations from our earlier study. In that work, the stable spirals were seen to exhibit very complex structures under the influence of straining effect $(\gamma \ne 0)$ such as drifting (meandering) or breaking up of the spiral wave, which is very similar to the phenomenon like monomorphic VT changes to polymorphic VT or ventricular fibrillation respectively. Furthermore, based on the characteristics of spiral wave patterns under the effect of straining, we had characterized the range of $\gamma$ into three distinct regimes: no break-up for $|\gamma | \le 0.01$, transitional $0.015\le |\gamma | \le 0.115$, and break-up regime $|\gamma | \ge 0.12$. In the no break-up regime, the spiral waves settle into periodic motions after the initial drift. In contrast, in the transitional regime, the spiral waves ended up as repeating island-type structures after initial drift and deformation. On the other hand, in the break-up regime, the spiral wave exhibited chaotic phenomena, which eventually resulted in the break-up of the spiral waves.

As in the previous section, initially we allow simulation to continue till $t=100$ without the presence of any obstacle or the medium motion ($\gamma =0$). Then we introduce the medium motion ($\gamma \ne 0$) and place the circular obstacle in the core region simultaneously and continue computation till a final time $t=1500$ is reached. Note that we consider only those values of strain rate $\gamma$ for which the spiral waves were seen to settle into periodic motions in the absence of an obstacle in our earlier study^29^, i.e., $|\gamma | \le 0.01$. More precisely, we choose the values $0.005,~-0.005,~0.01,~-0.01$.

#### The periodic regime

**Figure 8:**
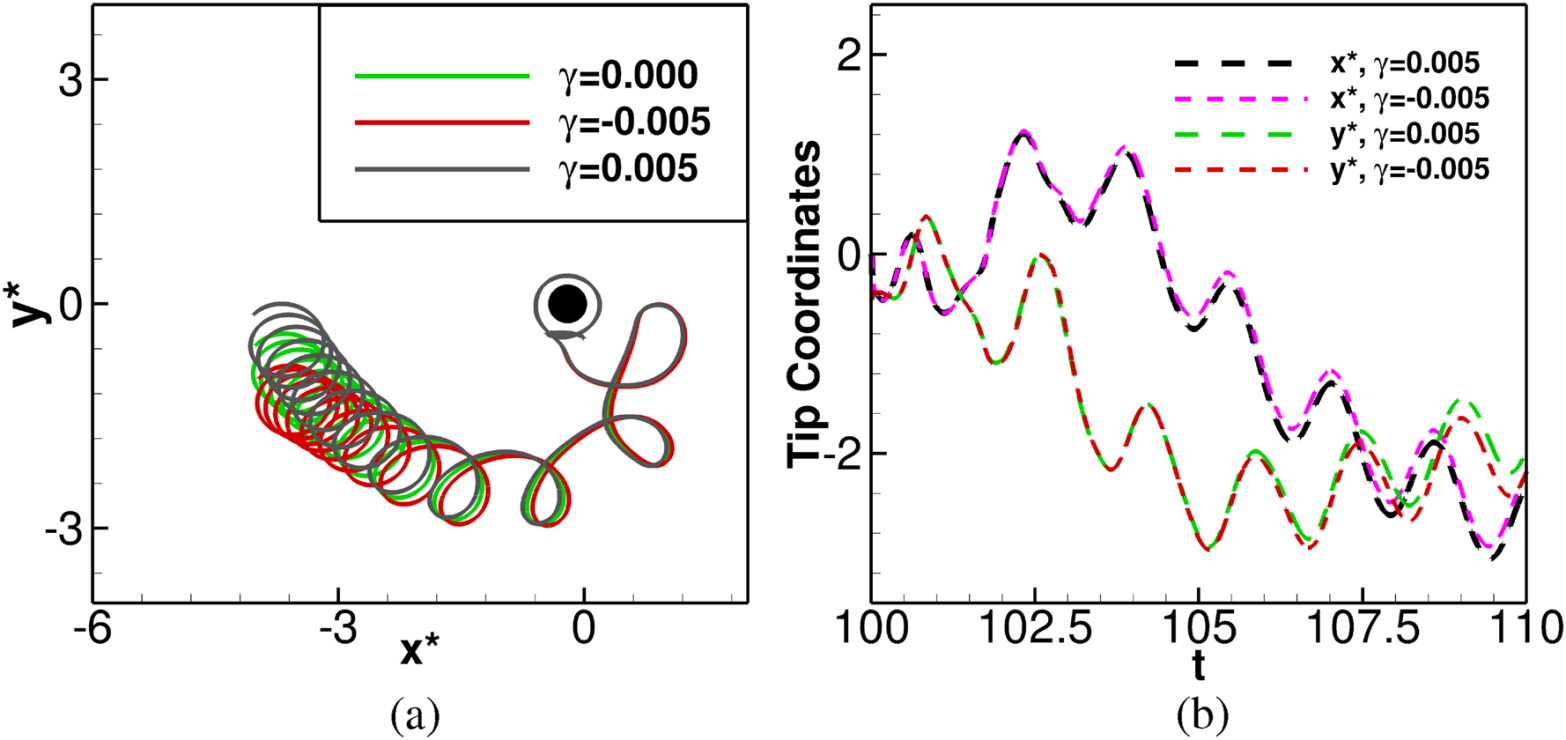
(**a**) Tip paths for $\gamma =0.000,~-0.005,~0.005$, with obstacle for $100\le t \le 120$, (**b**) time history of $(x^*, y^*)$ in $100\le t \le 110$ for $\gamma =-0.005,~0.005$, with the obstacle.

**Figure 9:**
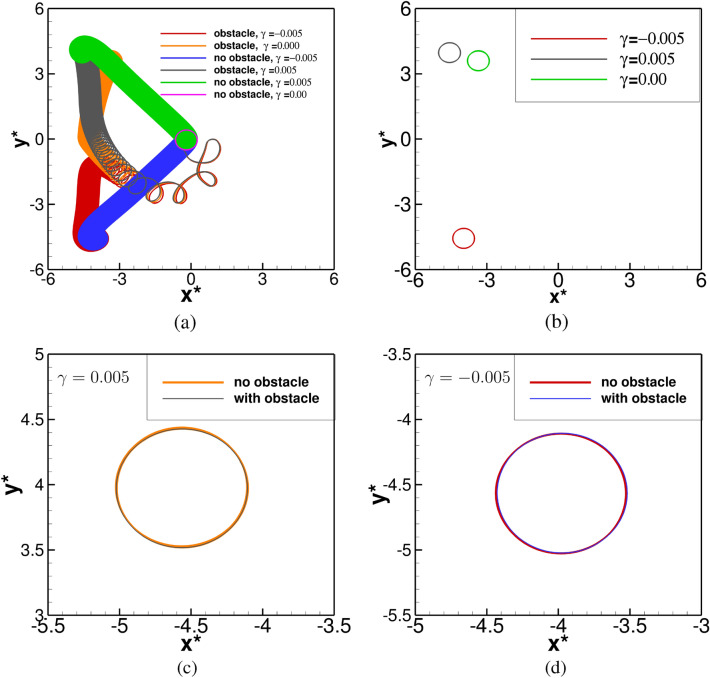
(**a**) Tip paths for $\gamma =0.000,~-0.005,~0.005$, with and without the obstacle, in $100\le t\le 1500$, (**b**) tip paths for $\gamma =0.000,~-0.005,~0.005$ with obstacle in $1100\le t\le 1200$, (**c, d**) tip paths with and without the obstacle in $1100\le t\le 1200$, for $\gamma =0.005$ and $-0.005$ respectively.

**Figure 10:**
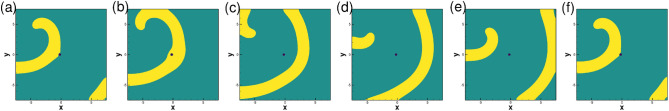
Evolution of periodic pattern under the effect of both ostacle and straining $(\gamma =0.005)$: (**a**) $t_0$, (**b**) $t_0+1*T/5$, (**c**) $t_0+2*T/5$, (**d**) $t_0+3*T/5$, (**e**) $t_0+4*T/5$, and (**f**) $t_0+5*T/5$. Here, $T=1.538$.

We present our results for $|\gamma |=0.005$ in Figs. 8-10. From Fig. 8a, which shows the early evolution of the tip trajectories in the time range $101.0001\le t \le 120$, it is clear that the spiral waves start drifting away from the circular obstacle after making one complete revolution around the circular obstacle (achieved around $t=101.40$), and for the earliest part of evolution, the tips follow the same trajectories, albeit, without the drain effect ($\gamma =0$) as well. This is reaffirmed by the time history plots of tip coordinates $(x^*, y^*)$ shown in the Fig. 8b, where tip coordinates can be seen following the same curves approximately up to $t=108.00$.

The long time evolution ($100\le t \le 1500$) of the trajectories can be seen from 9(a) where we have included the trajectories without the presence of the obstacle for the same values of $\gamma =-0.005\;\mathrm{and}\;0.005$ as well. Unlike the spiral waves without the obstacle being present, where they were seen to deform and stretch along the the principal directions $(-1,1)$ for $\gamma >0$, and $(-1,-1)$ for $\gamma <0$^29^ (blue and yellow color curves), they initially travel right to the obstacle for a very short span before moving towards the left side of the domain. During this period, the tip paths form some recognizable petal shape as can be seen from Figs. 8a and 9a. After that, they start deviating from each other and travel towards the different corners of the domain due to the straining effect. While the spiral wave moves towards the top left corner for $\gamma =0.005$, , it travels to the bottom left corner for $\gamma =-0.005$. At a later time, the spiral waves eventually settle into periodic motions for both the values of $\gamma =0.005,~ -0.005$ in the presence of the obstacle as can be seen from the closed circular trajectories in the phase portrait of the tip coordinates $(x^*, y^*)$ in Fig. 9b for $1100\le t\le 1200$.

More interestingly, depending on the sign of $\gamma$, the spiral waves exhibits similar behaviour asymptotically under the straining effect for $|\gamma |=0.005$ either with or without the presence of the obstacle which is asserted by Figs. 9c and 9d, where we have demonstrated the phase portraits of the tip coordinates $(x^*,y^*)$ for $1100 \le t \le 1200$. Once again, a fast Fourier transform of the time histories of the tip coordinates yields a single mode of dominant frequency at 0.650001. We show the patterns corresponding to one complete periodic cycle with time period $T=1.538$ for $\gamma =0.005$ at six time stations in Fig. 10a–f. Here one can see the spiral wave laterally hitting the obstacle (blue spot, Fig. 10a), which results in its swelling around the obstacle (Fig. 10b). Once the spiral wave gets detached from the obstacle, it regains uniform thickness (Fig. 10c) and evolves further Fig. 10d–f). One can see that Figs. 10a, f are exact replica of each other.

**Figure 11:**
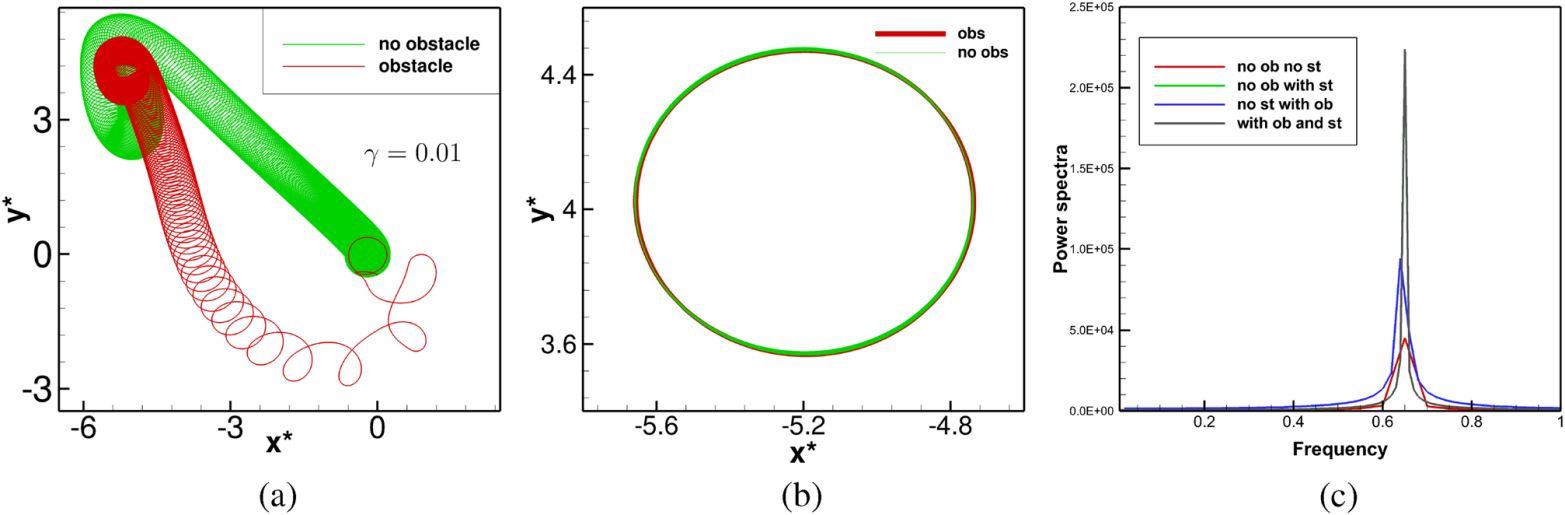
Tip trajectories of the spiral wave for $\gamma =0.01$ with and without the obstacle in the time range: (**a**) $100\le t\le 1500$, (**b**) $1100\le t\le 1200$ and, (**c**) power spectra of the time history of the *y*-coordinate of the tip trajectories for four different scenarios.

Next, we raise the value of $|\gamma |$ further to 0.01. For $\gamma =0.01$, the evolution of the spiral waves, with and without the obstacle are very similar to the ones obtained from the simulations corresponding to $\gamma =0.005$. This can be observed from Fig. 11a, b, where one can see the tip trajectory in the $x^*y^*$-phase plane finally settling into a periodic motion with the same time period $T=1.538$ towards the top-left corner of the domain. Note that for all the cases considered till now under the effect of straining, the spiral waves settle into periodic motion faster in presence of obstacle than without it. For example, for $\gamma =0.01$, the spiral wave attains periodicity around time $t=800$, in presence of an obstacle, while without it, it becomes periodic only after $t=1000$. This can be attributed to the combined effect of the straining and the obstacle, which introduces a perturbation into the system that triggers an early periodic motion of the spiral waves. One can clearly see from Figs. 8a, 9a and 11a that the presence of the obstacle facilitates the petal formation of the trajectories, which results in a much faster exit of the tip from the neighbourhood of the obstacle. This allows the trajectory to eventually settle into a closed orbit much faster than a trajectory without the presence of an obstacle. Also, the size of cores is independent of the strain rate or the presence of the obstacle. However, the introduction of medium motion displaces the cores (see Figs. 4b, 6b, 9b–d) to different locations.

For all the cases considered till now, which eventually yielded periodic motion of the spiral waves, we plot the power spectra of the time history of the *y*-coordinate of the tip trajectory for four individual cases in Fig. 11c: (i) no obstacle and no strain, (ii) with obstacle and no strain, (ii) no obstacle with strain and (iv) with both obstacle and strain. This figure reveals an interesting phenomenon pertaining to the energy content of the systems. One can clearly see from the spectral density or the amplitudes of the dominant frequency that the introduction of the obstacle causes an increase in the energy content which in turn increases multi-fold with the addition of medium motion.

#### The non-periodic regime

**Figure 12:**
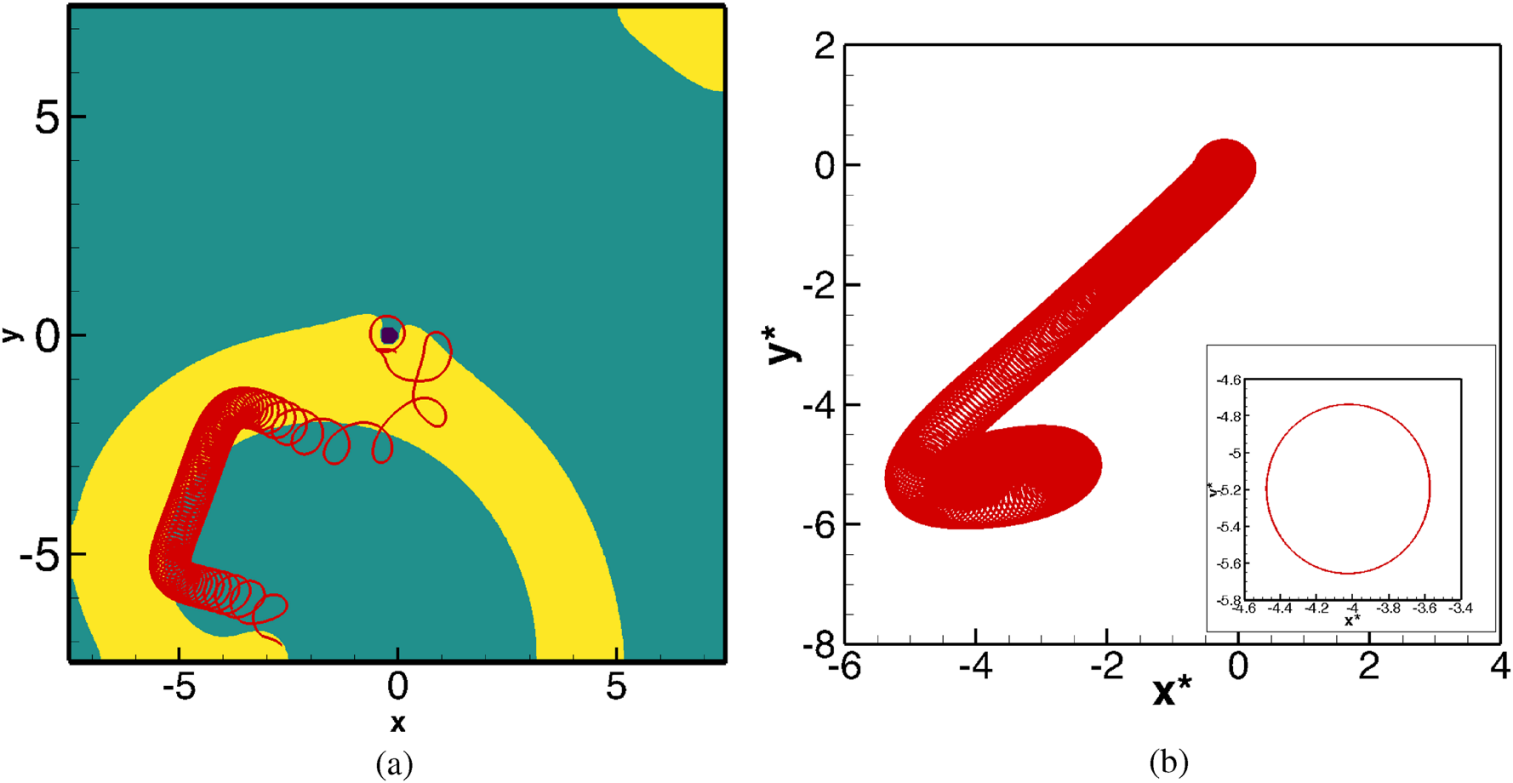
Dynamics for $\gamma =-0.01$: (**a**) tip path with the obstacle for $100\le t\le 216.11$ along with pattern at 216.10, (**b**) tip paths without the obstacle for $100\le t\le 1500$ with the final periodic pattern at the inset for $1200\le t\le 1300$.

**Figure 13:**
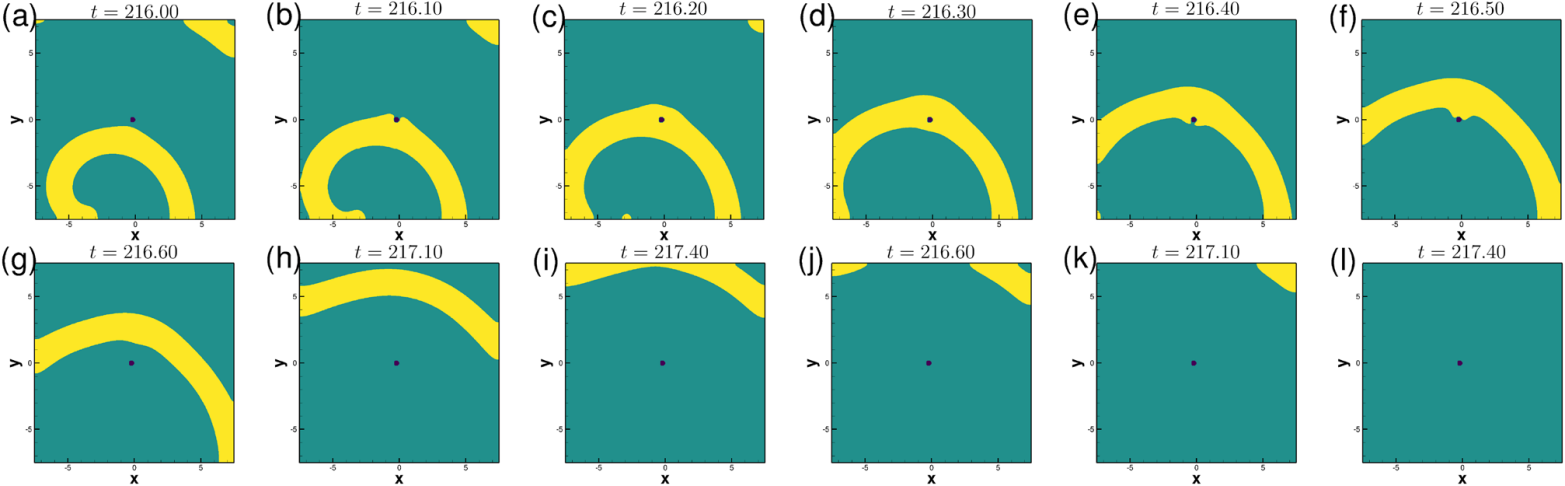
Spiral wave pattern evolutions for $\gamma =-0.01$, with a circular obstacle (blue spot), showing the eventual elimination of spiral wave from computational domain.

**Figure 14:**
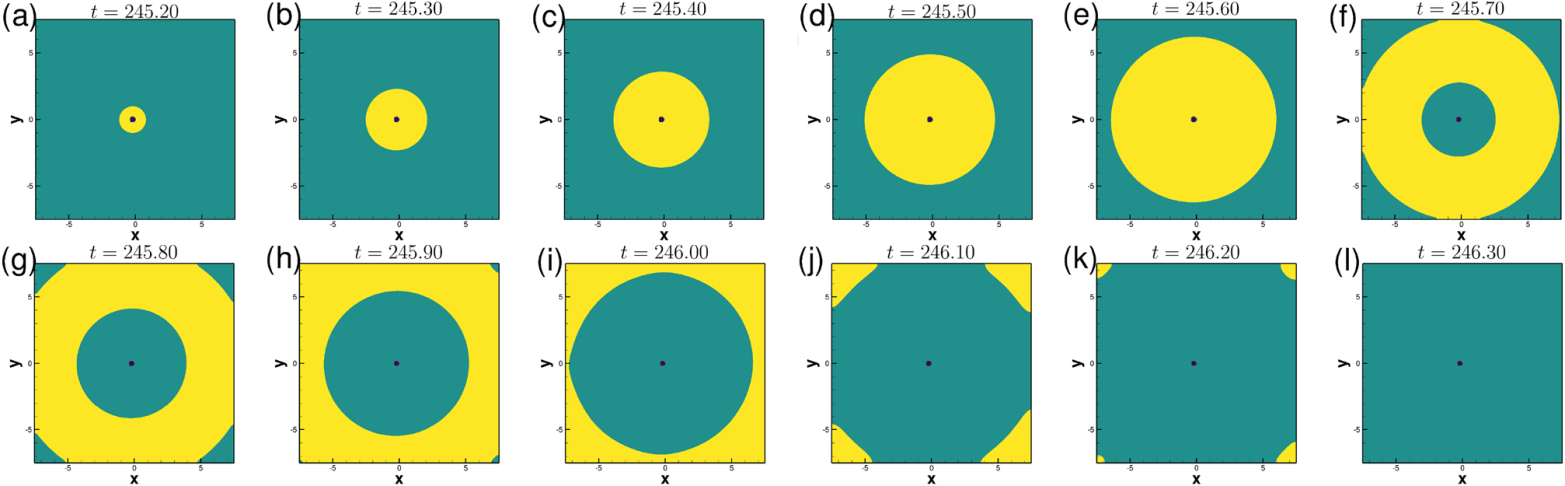
Pattern evolution for $\gamma =-0.01$ with the circular obstacle at a later time between $245.20\le t \le 246.30$ with a time increment $\Delta t=0.1$ between two consecutive patterns.

**Figure 15:**
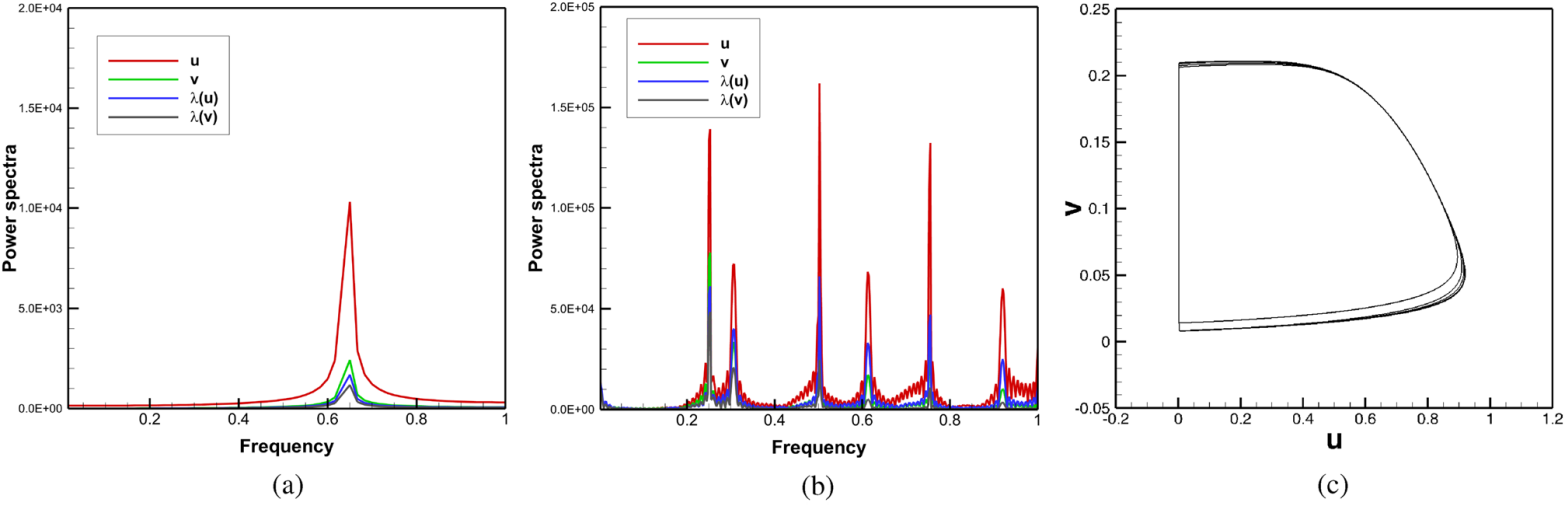
Power spectra of the local variables $u,\;v$ and order parameters $\lambda (u),\;\lambda (v)$ for the spiral wave in exciting media with circular obstacle for: (**a**) $\gamma =0$ in the time range $1400\le t\le 1500$, (**b**) $\gamma =-0.01$ in the time range $800\le t\le 1200$ and (**c**) phase portrait of the local variables $u,\;v$ corresponding to (**b**).

Till now, for all the parameters under consideration, the spiral waves under the influence of straining and the presence of a circular obstacle eventually settled into periodic motions. However, changing the value of $\gamma$ from 0.01 to $-0.01$ brings about a remarkable change in the behaviour of the patterns as depicted in Figs. 12–15. In Fig. 12a, we show the spiral wave pattern at $t=216.1$ along with the tip trajectory for $100\le t\le 216.10$. Although the initial movement of the tip is similar to the cases discussed in “The periodic regime” section, it eventually leaves the computational domain after tracing out the path as in Fig. 12a. On the other hand, for the same value of $\gamma$ without the presence of an obstacle, the spiral wave finally becomes periodic as Fig. 12b reveals.

The subsequent motion of the spiral waves for $\gamma =-0.01$ in the presence of the obstacle is shown in Fig. 13a–l. After exhibiting the patterns shown in the Fig. 13a–c, one can see the spiral wave tip leaving the domain through the zero flux bottom boundary at around time $t=216.30$, (see Fig. 13)d. The rest of the spiral wave, known as residual excited state^54^, travels toward the other boundaries of the domain (see Fig. 13e–j), and is followed by the complete elimination of the wave from the computational domain at $t=217.40$ through top right corner (see Fig. 13k–l). Interestingly, just as in the case of $\gamma =0.005$ in Fig. 10, the spiral wave, after laterally hitting the obstacle (Fig. 13a at time $t=216.1$) gains thickness around it (Fig. 13c–f) and once again regains its uniform thickness (Fig. 13h) before leaving the domain. For a more clear view, one may refer to the acompanying video “ellimination.avi” for the time interval $216.00\le t\le 218.00$.

For some times, no wave is visible in the computational domain. However, after some times, they re-enter the domain in a completely different shape as shown in Fig. 14a–l. High concentration wave-fronts start emerging around the circular obstacle in the form of a small disc (see Fig. 14a) at $t=245.2$. As time advances the size of it increases till time $t=245.60$ (see Fig. 14b–e). At the subsequent time 246.70 (see Fig. 14f), this wave-front is seen to convert into an annulus shape after detaching from the circular obstacle. This annulus-shaped wave keeps expanding in the radial direction (Fig. 14g–k) and it eventually leaves the computational domain at time 246.30 (Fig. 14l). For a more comprehensive view, one may refer to the accompanying video titled “stable1.avi”, where we have made a video for two consecutive cycles in time range $245\le t\le 248$. Remarkably, similar patterns are seen to repeat at later times also, depicted in the accompanying video “stable2.avi” for $802.70\le t\le 806.50$. Note that the thickness of the waves at these junctures are much larger than the ones described earlier.

As the spiral tip leaves the computational domain at $t=216.1$ (Figs. 12a, 13b), it is difficult to assert whether the motion of the spiral wave is periodic or not by analysing the time history of the tip data. In order to overcome this, we have adopted a slightly different strategy. Instead of monitoring the time history of the tip coordinates, we tracked the time history of *u* and *v* at the point $(-3.75, -3.75)$. As this pertains to analysing the dynamical behaviour of the spiral waves locally, to study its global behaviour, we further define an order parameter^55^, $\lambda (\phi )={\mathrm{max}}(\phi )\times {\mathrm{av}}(\phi )$ where ${\mathrm{max}}(\phi )$ and ${\mathrm{av}}(\phi )$ are the maximum and average values of the state variable $\phi$ respectively in the computational domain. To show that this approach captures the spiral wave dynamics accurately, firstly we show the power spectra of the time histories of the pairs $(u, \lambda (u))$ and $(v, \lambda (v))$ in Fig. 15a which corresponds to the trajectory shown in Fig. 4b. The presence of a single dominant frequency for both the pairs $(u, \lambda (u))$ and $(v, \lambda (v))$ corresponding to the value 0.65001 establishes the validity of our approach as the power spectra analysis of the time history of the tip coordinates led to the same conclusion. Next, in Fig. 15b, we present the power spectra for the same pairs for $\gamma =-0.01$ in the time range $800\le t\le 1200$. The existence of multiple modes of the dominant frequencies clearly demonstrates that the spiral waves do not settle into periodic motion in this case and in turn, show signs of a transitional phase. The phase portrait of the local variables *u* and *v* shown in Fig. 15c reconfirms this fact. Note that without the circular obstacle, $|\gamma |=0.01$ yielded only a periodic motion of the spiral wave and thus the role of the obstacle is to trigger an early passage to transitional phase for a much lower strain rate.

As mentioned in the introduction section, the transition of a spiral wave from periodic to drifting (or meandering) motion in cardiac tissue may be the hallmark of the transition of monomorphic VT to polymorphic VT, which has already been conjectured by numerous experimental and numerical studies^1,17–19^. These studies further concluded that VT, which is always preceded by ventricular fibrillation is the most frequent cause of fatalities. Also, the transition of a meandering (drifting) spiral wave to spiral wave breakup may correspond to the transition of polymorphic VT to ventricular fibrillation, resulting in sudden cardiac death. This is where the observations from our study could be relevant and of practical importance.

## Conclusions

The effect of a circular obstacle on the dynamics of rigidly rotating spiral waves has been investigated with and without the presence of medium motion in the present study. We employ a recently reconstructed spatially fourth and temporally second order accurate, implicit, unconditionally stable high order compact scheme to carry out numerical simulations of the Oregonator model of excitable media. Firstly, the spiral wave is allowed to settle into a periodic motion in isolation without the presence of the obstacle and medium motion. The circular obstacle is placed in the core region and the role of the stoichiometric parameter on the propagation of the ensuing waves is thoroughly examined. The tip of the waves were seen to drift away from the obstacle and settle into periodic motion by relocating the cores at different corners of the computational domain depending on the parameter value.

Next, we investigated the combined effect of a circular obstacle and the medium motion (straining) on the dynamics of periodically rotating spiral waves the stoichiometric parameter value 1.4. To the best of our knowledge, this is probably the first time such study has been undertaken in the existing literature. Numerical simulations are carried out for the straining rates $\gamma =0.005,\;-0.005,\; 0.01\;\mathrm{and}\; -0.01$. In the presence of the obstacle, the tip trajectories after revolving around the obstacle for an extremely small period of time, drifts away from it and follow almost the same trajectory in the form of petals for time duration about 8.0. For $\gamma =0.005\;\mathrm{and}\;0.01$, they eventually settle into periodic orbits at the north-west and for $\gamma =-0.005$, at the south-west corner of the computational domain. The relocated core regions are the same as their no obstacle counterparts, which follow almost straight line trajectories towards the corner. Furthermore, the spiral waves in the presence of the obstacle settle into a periodic motion at a much earlier time than those without the obstacle. Overall, for the systems exhibiting periodic motion, the presence of the obstacle was seen to enhance their energy content with introduction of medium motion further inflating it multi-fold.

For $\gamma =-0.1$, the spiral wave patterns were found to be markedly complex and different from all previous studies in the current work. After displaying patterns similar to the other $\gamma$ values in the early part of the evolution, with passage of time, it was seen to inch towards the zero flux bottom boundary of the domain and subsequently disappear for some time. After a while, a high concentration wave-front in the form of a disc, was seen to appear around the obstacle, gradually increasing its size and eventually transfigure into an annulus form. This annulus expands radially and eventually gets eliminated from the domain colliding through its boundaries. Similar patterns have been observed to repeat at later times. While a power spectra analysis of the tip coordinates were used to establish periodicity in all the previous cases, use of a user defined order parameter reveals that for $\gamma =-0.1$, the system is leaning towards a transitional phase.

As mentioned earlier, experimental studies on the simultaneous effects of obstacle and medium motion is not very common in the scientific community. We strongly believe that the numerical results in the present paper have the potential to add value to future experimental studies on spiral wave dynamics, dealing with effects similar to that of medium motion and obstacle simultaneously. As such, we are extremely hopeful that the observations from our study would be able to garner enough interest amongst experimentalists into studying the effects of these two on cardiac arrhythmias. We are currently working on the effects of size and shape of the obstacle, the initial condition and size of the domain on the on the spiral waves with straining, which will be discussed in a separate study in the near future.

## Supplementary Information

Supplementary Information 1.

Supplementary Information 2.

Supplementary Information 3.

Supplementary Information 4.

## Data Availability

The data that support the findings of this study are available from the corresponding author upon reasonable request.
