# Supplementary material for: Influence of a circular obstacle on the dynamics of stable spiral waves with straining
Source: Sci Rep. 2022 Aug 25;12:14479. doi: 10.1038/s41598-022-18602-0 (PMC9411171; doi:10.1038/s41598-022-18602-0)
Supplement: Supplementary file 1 — Supplementary Information 1. [file 41598_2022_18602_MOESM1_ESM.doc]

**Descriptions of the videos attached as supplementary materials**

1: The video titled ‘ellimination.avi’ demonstrates the elimination of wavefront from the computational domain depicted in figure 13.

2: The video titled ‘stable1.avi’ illustrates the evolution of waves in the computational domain depicted in figure 14 for the time range t=245 to t=248.

3: The video titled ‘stable2.avi’ illustrates the evolution of waves in the computational domain depicted in figure 14 at a later time range of t=802.70 to t=806.50.
